# Development of edible Thai rice film fortified with ginger extract using microwave-assisted extraction for oral antimicrobial properties

**DOI:** 10.1038/s41598-021-94430-y

**Published:** 2021-07-21

**Authors:** Niramon Utama-ang, Sirinapa Sida, Phenphichar Wanachantararak, Arthitaya Kawee-ai

**Affiliations:** 1 Division of Product Development Technology, Faculty of Agro-Industry, Chiang Mai University, Chiang Mai, 50100 Thailand; 2 Cluster of High Value Product from Thai Rice and Plants for Health, Chiang Mai University, Chiang Mai, 50100 Thailand; 3 Research Center for Development of Local Lanna Rice and Rice Product, Chiang Mai University, Chiang Mai, 50200 Thailand; 4 Faculty of Dentistry, Chiang Mai University, Chiang Mai, 50200 Thailand

**Keywords:** Biochemistry, Biotechnology, Microbiology, Health care

## Abstract

This study aimed to investigate microwave-assisted extraction (MAE) of dried ginger and to develop a rice-based edible film incorporating ginger extract. The optimal MAE conditions of 400 W microwave power and an extraction time of 1 min were determined using a 3^2^ full factorial design. The optimized extract showed total phenolic compounds (TPC, 198.2 ± 0.7 mg gallic acid equivalent/g), antioxidant activity measured by DPPH (91.4 ± 0.6% inhibition), ABTS (106.4 ± 3.1 mg Trolox/g), and FRAP (304.6 ± 5.5 mg Trolox/g), and bioactive compounds including 6-gingerol (71.5 ± 3.6 mg/g), 6-shogaol (12.5 ± 1.0 mg/g), paradol (23.1 ± 1.1 mg/g), and zingerone (5.0 ± 0.3 mg/g). Crude extract of dried ginger showed antimicrobial activity against *Streptococcus mutans* DMST 18777, with a minimum inhibitory concentration and minimum bactericidal concentration of 0.5 and 31.2 mg/mL, respectively. The rice-based edible film incorporating 3.2% (w/v) ginger extract tested against *S. mutans* DMST 18777 had a mean zone of inhibition of 12.7 ± 0.1 mm. Four main phenolic compounds, 6-gingerol, 6-shogaol, paradol, and zingerone, and six volatile compounds, α-curcumene, α-zingiberene, γ-muurolene, α-farnesene, β-bisabolene, and β-sesquiphellandrene, were found in rice film fortified with crude ginger extract.

## Introduction

Recently, the incorporation of natural additives from plant extracts in edible films or coatings has gained increasing attention among researchers. Natural additives have been applied to improve texture, rheology, and functional properties such as antioxidant, antimicrobial, and anti-browning properties of edible films^1^. Rice (*Oryza sativa* L.) is one of the main foods for almost half of the global population. Rice flour is widely used as a food hydrocolloid due to its low cost, convenience, biodegradability, and easy processability^2^. Furthermore, rice flour can be formulated with other components to improve the physical, chemical, sensory, and nutritional properties of the product^3^. Films or coatings produced with rice flour are generally transparent, odorless, colorless, and tasteless^2^.

*Zingiber officinale* Roscoe (ginger), a member of the Zingiberaceae family, is commonly used as a spice in food and as a traditional medicine in Asian countries. Many bioactive compounds of ginger such as phenolic compounds (gingerols, shogerols, and paradols), terpenes (zingiberene, β-bisabolene, and α-curcumene), polysaccharides, lipids, and organic compounds are known to have biological activity, for example, antioxidant, anticancer, anti-diabetic, anti-inflammatory, antimicrobial, and cardiovascular protective activity^4^. However, conventional methods for extracting the bioactive compounds require a long extraction time and affect the quality of the final product by causing the loss of some of the volatile compounds, which leads to low extraction efficiency^5^. The microwave-assisted technique has been widely employed to extract phenolic compounds from plants due to its small equipment size, simplicity, and rapidness. The efficiency of microwave-assisted extraction (MAE) has been found to be two times higher than that of the conventional method^6,7^.

In a previous study, 9 month-matured ginger dried at 60 °C for 308 min presented the highest content of total phenolic compounds (TPC, 12.2 µmol tannic acid/g) and 6-gingerol (12.5 mg/g)^8^. The nonvolatile compounds including gingerols, shogaols, paradols, and zingerone in ginger exhibit antioxidant activity; dried and roasted ginger has a high content of shogaols, paradols, and zingerone^9^. Ginger is known to harden the teeth because of indirect mineralization properties, thus it has been validated for oral care^10^. We aimed to develop an edible film from rice with an antimicrobial effect. There is no information about the effect of the inclusion of crude ginger extract on rice film properties. Thus, the first objective was to investigate the effect of MAE conditions (microwave power and extraction time) on the yield, antioxidant activity, and bioactive compounds of dried ginger. The second objective was to integrate crude ginger extract into rice film and to determine its antimicrobial activity, antioxidant activity, and bioactive compounds.

## Results

### Effect of microwave power and extraction time on ginger extraction yield

**Figure 1:**
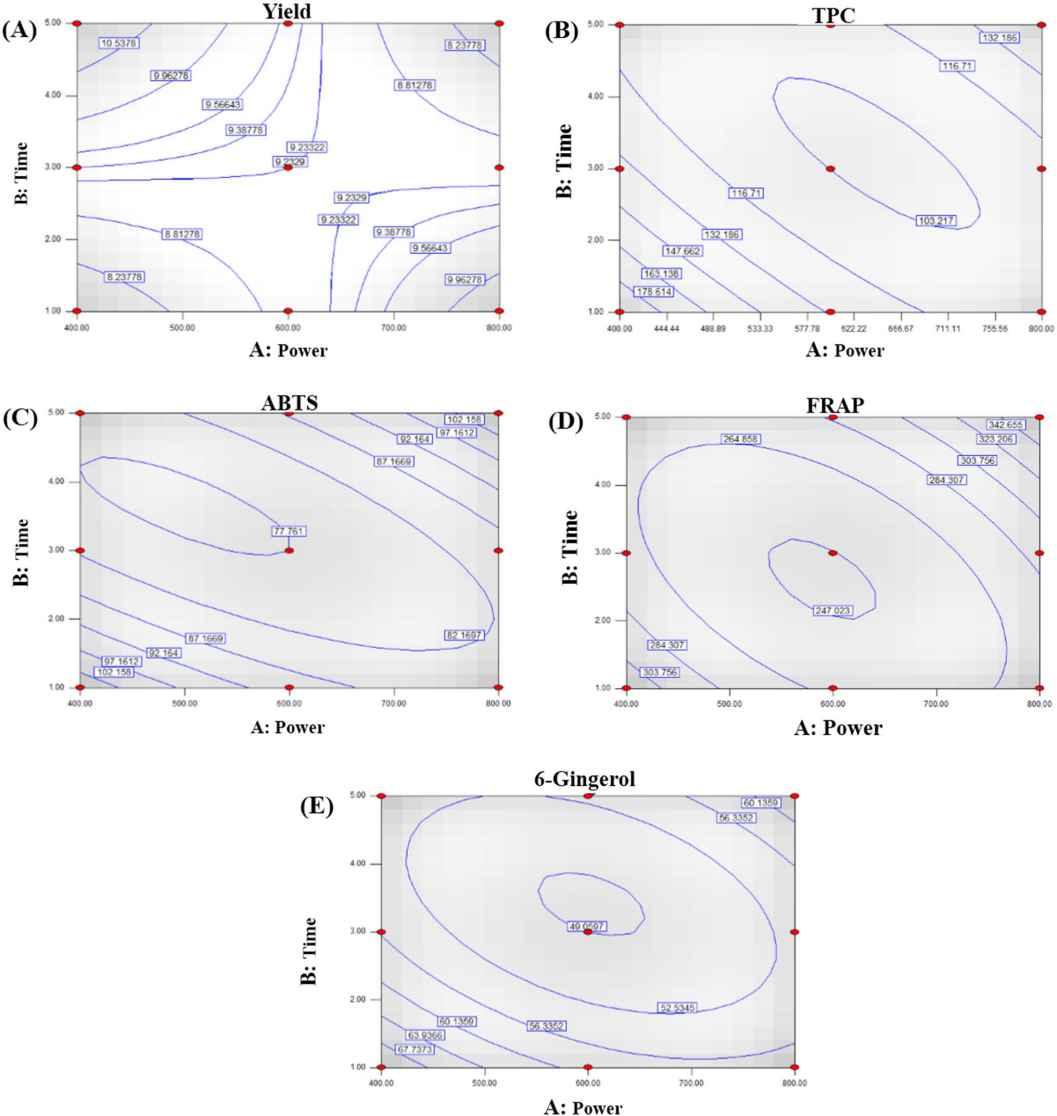
Contour plots of the effect of the interaction between power and time on (**A**) yield, (**B**) TPC, (**C**) ABTS, (**D**) FRAP, and (**E**) 6-gingerol.

To define the effect of MAE, the microwave power (400, 600, and 800 W) and time (1, 3, and 5 min) were set to determine the extraction yield, TPC, antioxidant activity (determined by DPPH, ABTS, and FRAP assays), and content of 6-gingerol, 6-shogaol, paradol, and zingerone of dried ginger (Table 1). There was a significant improvement in extraction yield with an increase of microwave power from 400 to 800 W; however, the yield decreased with a further increase in extraction time (*p* ≤ 0.05). The yield of crude extract was approximately 7.3 ± 1.0 to 10.7 ± 1.3% dry weight (DW). The regression equation of the yield was fitted with a coefficient of determination (*R*^2^) of 0.7044 (Table 2). Extraction yield showed a positive correlation with microwave power and time; however, the combination of microwave power and extraction time showed a negative effect, which was correlated with the contour plot (Fig. 1A). The yield of crude extract initially increased with an increase in microwave power and extraction time. However, increasing the microwave power to more than 600 W resulted in a decrease in the extraction yield.

**Table 1:** Operation parameters and responses for microwave-assisted ginger extraction by 3^2^ full factorial design.

Microwave		Yield (%, DW)*,**	TPC (mg GAE/g)	Antioxidant activity			Bioactive compounds			
Power (W)	Time (min)			DPPH (%inhibition)	ABTS (mgTrolox/g)	FRAP (mgTrolox/g)	6-Gingerol (mg/g)	6-Shogaol (mg/g)	Paradol (mg/g)	Zingerone (mg/g)
400	1	7.5 ± 0.5^b^	200.8 ± 4.5^a^	91.8 ± 0.4^a^	104.2 ± 3.0^b^	297.3 ± 13.1^b^	73.4 ± 1.3^a^	12.8 ± 0.8^bc^	25.5 ± 7.8^a^	4.9 ± 0.2^bc^
600	1	9.3 ± 1.2^a^	119.9 ± 5.6^d^	89.2 ± 0.9^b^	93.3 ± 4.5^c^	296.6 ± 2.7^b^	60.4 ± 2.4 ^bc^	12.5 ± 1.8^bc^	23.5 ± 0.8^a^	5.1 ± 0.1^b^
800	1	10.2 ± 0.4^a^	111.3 ± 5.4^e^	81.5 ± 2.4^e^	81.0 ± 5.9^de^	267.9 ± 1.4^d^	56.1 ± 1.2^bcd^	10.9 ± 0.6^bc^	17.9 ± 0.6^ab^	4.6 ± 0.2^ cd^
400	3	9.9 ± 0.5^a^	132.2 ± 5.4^c^	84.3 ± 0.2^d^	83.4 ± 7.3^d^	278.4 ± 2.3^c^	56.4 ± 1.3^bcd^	11.9 ± 0.2^bc^	19.9 ± 2.9^ab^	4.5 ± 0.0^cde^
600	3	10.4 ± 0.1^a^	104.9 ± 0.9^e^	89.2 ± 0.9^b^	72.6 ± 3.7f.	245.9 ± 2.5^e^	47.2 ± 1.3^d^	9.9 ± 1.9^c^	23.7 ± 1.42^a^	4.3 ± 0.1^de^
800	3	9.7 ± 0.1^a^	109.8 ± 1.0^e^	85.8 ± 0.5^ cd^	86.6 ± 2.2^ cd^	282.2 ± 1.8^c^	54.3 ± 1.8^bcd^	11.8 ± 2.0^bc^	16.6 ± 1.1^b^	4.3 ± 0.1^de^
400	5	10.4 ± 0.7^a^	111.1 ± 1.3^e^	85.8 ± 0.2^ cd^	87.3 ± 5.0^ cd^	284.0 ± 2.6^c^	57.4 ± 2.1^bcd^	13.1 ± 0.7^b^	20.4 ± 1.21^ab^	4.9 ± 0.3^bc^
600	5	10.7 ± 1.3^a^	119.8 ± 5.4^d^	86.8 ± 1.2^ cd^	74.6 ± 1.8^ef^	266.9 ± 2.2^d^	51.1 ± 1.6^ cd^	10.3 ± 0.8^bc^	16.6 ± 2.0^b^	4.1 ± 0.5^e^
800	5	7.3 ± 1.0^b^	145.3 ± 1.8^b^	89.5 ± 0.7^b^	120.9 ± 2.3^a^	377.8 ± 4.3^a^	64.7 ± 2.5^ab^	16.0 ± 1.8^a^	19.3 ± 1.3^ab^	5.8 ± 0.0^a^

*DW means dry weight.

**a-f represented the significant difference in the columns at *p* < 0.05.

**Table 2:** Regression coefficients of the models for extraction yield, TPC, ABTS, FRAP, and 6-gingerol.

Response	Final equation in terms of actual factors	*R*^2^	*P-*value
Extraction yield (%)	+ 2.6 + 0.01 * Power^ns^ + 2.3 * Time^ns^ − 3.6 × 10^−3^ * Power * Time	0.7044	0.0498
TPC (mg GAE/g)	+ 512.1 − 0.9 * Power − 76.1 * Time + 5.0 × 10^–4^ * Power^2^ + 4.3 * Time^2^ + 0.07 * Power * Time	0.9758	0.0025
ABTS (mgTrolox/g)	+ 205.3 − 0.2 * Power^ns^ − 35.4 * Time^ns^ + 1.4 × 10^–4^ * Power^2;ns^ + 2.7 * Time^2^ + 0.03 * Power * Time	0.9267	0.0218
FRAP (mgTrolox/g)	+ 710.6 − 1.2 * Power^ns^ − 82.8 * Time^ns^ + 8.5 × 10^–4^ * Power^2^ + 6.7 * Time^2,ns^ + 0.08 * Power * Time	0.9005	0.0391
6-Gingerol (mg/g)	+ 148.8 − 0.2 * Power^ns^ − 20.1 * Time^ns^ + 1.4 × 10^−4^ * Power^2^ + 1.7 * Time^2^ + 0.01 * Power * Time	0.9267	0.0218

*ns means not significant at *p* < 0.05.

### Effect of microwave power and extraction time on TPC and antioxidant activity

Conversely, TPC and antioxidant activity measured by DPPH, ABTS, and FRAP methods decreased significantly with an increase of both power and time (*p* ≤ 0.05, Table 1). The highest values for TPC and DPPH activity were 200.8 ± 4.5 mg gallic acid equivalent (GAE)/g and 91.8 ± 0.4%, respectively, at MAE conditions of 400 W and 1 min, while ABTS and FRAP values of 120.9 ± 2.3 and 377.8 ± 4.3 mg/g, respectively, were obtained when 800 W of microwave power was applied for 5 min. The summary of the analysis of variance (ANOVA) representing the results indicated that TPC, ABTS, and FRAP values were reliable and significant (*p* < 0.05) with *R*^2^ values of 0.9758, 0.9267, and 0.9005, respectively (Table 2). Microwave power and extraction time were found to be non-significant (*p* > 0.05) for these responses, which means that an increase of microwave power and extraction time decreased the TPC, ABTS, and FRAP values. However, the interaction of microwave power and extraction time had a positive effect on the values observed. Consistent with the ANOVA results, MAE presented a negative effect for TPC (Fig. 1B), ABTS (Fig. 1C), and FRAP (Fig. 1D), with a significant decrease in their values when high microwave power (> 500 W) and a long extraction time (> 2 min) were applied.

### Effect of microwave power and extraction time on bioactive compounds

The highest yield of 6-gingerol was obtained either with low microwave power and a short extraction time (400 W, 1 min) or high microwave power and a long extraction time (800 W, 5 min). The highest paradol content was produced when microwave power was in the range 400–600 W, while the highest yield of 6-shogaol and zingerone was achieved when microwave power was 800 W and extraction time was 5 min. The highest values for 6-gingerol and paradol (73.4 ± 1.3 and 25.5 ± 7.8 mg/g, respectively) were obtained at low power (400 W, 1 min), while the highest values for 6-shogaol and zingerone were obtained at high power and a longer extraction time (800 W, 5 min) with values of 16.0 ± 1.8 and 5.8 ± 0.0 mg/g, respectively (Table 1). However, a model for using MAE to extract compounds for ginger could only be generated for 6-gingerol (*p* < 0.05, Table 2). A high *R*^2^ value (0.9267) was achieved and microwave power and extraction time were found to be non-significant (*p* > 0.05). Consistent with the ANOVA and contour plots for TPC, ABTS, and FRAP, MAE had a negative effect for 6-gingerol, their values decreasing significantly when high microwave power (> 500 W) and a long extraction time (> 2 min) were used (Fig. 1E).

### Optimization and validation of MAE conditions

**Figure 2:**
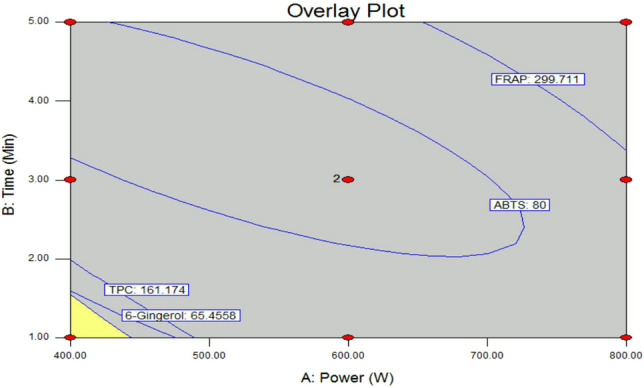
Overlay plot for the optimal conditions for microwave-assisted ginger extraction.

The contour response surfaces were plotted to study the interactions between the factors for the significant responses to determine the optimum levels of each factor required to obtain maximum yields of TPC, ABTS, FRAP, and 6-gingerol (Fig. 2). Based on the analysis and calculation, the validation experiment was conducted with microwave power of 400 W and an extraction time of 1 min. The yield of crude ginger (7.6 ± 0.6%) was close to the predicted value of 7.6%. TPC and antioxidant activity indicated by ABTS and FRAP values were 198.2 ± 0.7 mg GAE/g, 106.4 ± 3.1 mg Trolox/g, and 304.6 ± 5.5 mg Trolox/g, respectively (Table 3). The yield of 6-gingerol was 71.5 ± 3.6 mg/g. The approximate error between predicted and experimental values was in the range − 7.1 to 2.6%, within ± 10% and so indicating that the validation results were acceptable and consistent with the predicted values.

**Table 3:** Predicted and actual values for optimal conditions.

Response	Actaual value	Predicted value	% Error
Yield (%)	7.6 ± 0.6	7.6	0.6
TPC (mg GAE/g)	198.2 ± 0.7	193.9	2.1
DPPH (%)	91.4 ± 0.6	89.0	2.6
ABTS (mgTrolox/g)	106.4 ± 3.1	106.0	0.4
FRAP (mgTrolox/g)	304.6 ± 5.5	317.9	− 4.3
6-Gingerol (mg/g)	71.5 ± 3.6	71.5	0.1
6-Shogaol (mg/g)	12.5 ± 1.0	12.7	− 2.1
Paradol (mg/g)	23.1 ± 1.1	24.7	− 7.1
Zingerone (mg/g)	5.0 ± 0.3	5.2	− 3.3

## Application of crude ginger extract in rice-based edible film

### Antimicrobial activity of rice-based edible film

The disc diffusion analysis results revealed that the crude extract of ginger (63–500 mg/mL) showed no significant difference on the inhibition zone (*p* < 0.05) in antimicrobial activity against *Streptococcus mutans* DMST 18777, with an average inhibition zone of 9.5 ± 0.7 to 11.0 ± 1.4 mm (Table 4). The minimum inhibitory concentration (MIC) and minimum bactericidal concentration (MBC) of the crude extract of ginger against *S. mutans* DMST 18777 were 0.5 and 31.2 mg/mL, respectively. Thus, the highest concentration of crude ginger extract that was incorporated into the rice-based edible film was 32 mg/mL (3.2%, w/v). Inhibition zone diameters for rice-based edible film disks with various concentrations of crude ginger extract (0, 4, 8, 16, and 32 mg/mL or 0.4%, 0.8%, 1.6%, and 3.2%, w/v) against *S. mutans* DMST 18777 are presented in Table 3. No inhibition was observed for rice film not containing crude ginger extract, indicating that rice film alone did not affect antimicrobial activity. The incorporation of less than 1.6% (w/v) crude ginger extract in the rice film was not enough to inhibit the growth of *S. mutans* DMST 18777. The most efficient concentration of ginger extract was 3.2% (w/v); film containing this amount had an inhibition zone of 12.7 ± 0 0.1 mm while tetracycline presented an inhibition zone of 41.2 ± 0.4 mm.

**Table 4:** Antimicrobial activity of crude ginger extracts and rice-based edible film at different concentrations against *S. mutans* by disc diffusion method.

Sample	Concentration (mg/mL)	Inhibition zone (mm)
DMSO (Negative control)		–
Crude ginger extract^ns^*	63	11.0 ± 1.4
	125	11.0 ± 0.0
	250	10.5 ± 0.7
	500	9.5 ± 0.7
MIC** (mg/mL)	0.5	
MBC** (mg/mL)	31.2	
DMSO (Negative control)		–
Tetracycline 30 µg (Positive control)		41.2 ± 0.4^a^
Rice film + ginger extract	0	–
	4.0	–
	8.0	–
	16.0	–
	32.0	12.7 ± 0.1^b^

*ns means not significantly different at *p* ≤ 0.05.

**MIC is Minimum inhibitory concentration; MBC is Minimum bactericidal concentration.

### Changes of TPC, antioxidant activity, bioactive compounds, and volatile compounds of rice film

The TPC, antioxidant activity, and bioactive compounds (6-gingerol, 6-shogaol, paradol, and zingerone) of the edible rice films increased gradually with an increase of crude ginger extract content (Table 5). Paradol and zingerone were absent from rice film containing 4.0 mg/mL of ginger extract. The main bioactive compounds present in the rice film were 6-gingerol followed by 6-shogaol, paradol, and zingerone, respectively. Meanwhile, the rice films showed no significant differences (*p* < 0.05) in the principal volatile compounds of crude ginger, including α-curcumene, α-zingiberene, γ-muurolene, α-farnesene, β-bisabolene, and β-sesquiphellandrene. The main compounds found in the rice film fortified with crude ginger extract were present in the order α-zingiberene > α-curcumene ≥ β-sesquiphellandrene > α-farnesene > γ-muurolene ≥ β-bisabolene. Rice film without ginger extract showed no TPC, antioxidant activity, bioactive compounds, or volatile compounds.

**Table 5:** Phenolic compounds, antioxidant activity, and volatile compounds in rice film strip incorporating various concentrations of crude ginger extract.

Investigate parameters	Unit	Ginger extract concentration in rice film (%, w/v)				
		0	0.4	0.8	1.6	3.2
TPC	mg GAE/100 g	–	415.8 ± 10.9^d,^**	559.4 ± 5.5^c^	992.7 ± 7.3^b^	1,613.3 ± 7.3^a^
Antioxidant activity		–				
DPPH	mgTrolox/ 100 g		392.0 ± 47.8^d^	939.1 ± 65.9^c^	1,732.9 ± 51.0^b^	2,637.8 ± 88.3^a^
ABTS	mgTrolox/ 100 g	–	347.0 ± 41.4^d^	671.7 ± 56.1^c^	865.04 ± 21.9^b^	1,634.7 ± 72.8^a^
FRAP	mgTrolox/ 100 g	–	1,060.2 ± 50.4^d^	2,137.8 ± 66.7^c^	3,346.6 ± 75.6^b^	6,244.6 ± 90.9^a^
Bioactive compounds						
6-Gingerol	mg/g	–	10.4 ± 0.0^c^	1.9 ± 0.1^c^	7.1 ± 0.1^b^	12.9 ± 0.3^a^
6-Shogaol	mg/g	–	0.5 ± 0.0^c^	0.6 ± 0.0^c^	2.1 ± 0.1^b^	5.5 ± 0.9^a^
Paradol	mg/g	–	ND	0.8 ± 0.0^c^	1.4 ± 0.0^b^	1.8 ± 0.1^a^
Zingerone	mg/g	–	ND	0.2 ± 0.0^c^	0.4 ± 0.0^b^	0.7 ± 0.0^a^
Volatile compounds						
α-Curcumene^ns^*	%	–	16.8 ± 2.9	13.2 ± 2.1	14.5 ± 2.9	13.8 ± 1.5
α-Zingiberene^ns^	%	–	27.0 ± 4.3	30.8 ± 4.8	33.2 ± 5.8	34.6 ± 6.7
γ-Muurolene^ns^	%	–	7.6 ± 1.2	6.3 ± 0.3	6.8 ± 1.0	6.5 ± 1.0
α-Farnesene^ns^	%	–	10.3 ± 1.6	10.5 ± 1.0	12.6 ± 2.6	12.7 ± 2.0
β-Bisabolene^ns^	%	–	8.7 ± 1.0	6.7 ± 1.0	6.6 ± 1.6	6.2 ± 2.2
β-Sesquiphellandrene^ns^	%	–	15.3 ± 2.4	14.1 ± 2.2	13.3 ± 3.9	13.1 ± 4.5

*ns means not significant at *p* < 0.05.

**a–d represented the significant difference in the rows at *p* < 0.05.

## Discussion

Generally, an increase of microwave power increased the extraction yield with a shorter extraction time^11^. The changes of antioxidant activity might be due to the generation of free radicals such as H^+^, OH^−^, and electrons through microwave radiation^4^. The highest yield of 6-gingerol was obtained either with low microwave power and a short extraction time (400 W, 1 min) or high microwave power and a long extraction time (800 W, 5 min) .This agrees with the results of Teng et al.^5^ who found that increasing the heating time increased the quantity of compounds extracted, while others found that a variation of microwave power from 500 to 1000 W had no significant effect on the flavonoid yields from ginger^12^. The negative effect of the interaction between microwave power and extraction time on extraction yield, bioactive compounds, and their antioxidant activity meant that these responses decreased significantly at the higher level of microwave power and prolonged extraction time^13^. The microwave irradiation time is influenced by the dielectric constant of the solvent, especially ethanol and methanol, and a prolonged irradiation time can result in overheating and the risk of losing thermolabile constituents, i.e. gingerols^14^. In this study, the optimal conditions for dried ginger were 400 W of microwave power and 1 min of extraction time, which protected the antioxidant compounds in ginger from thermal destruction.

**Figure 3:**
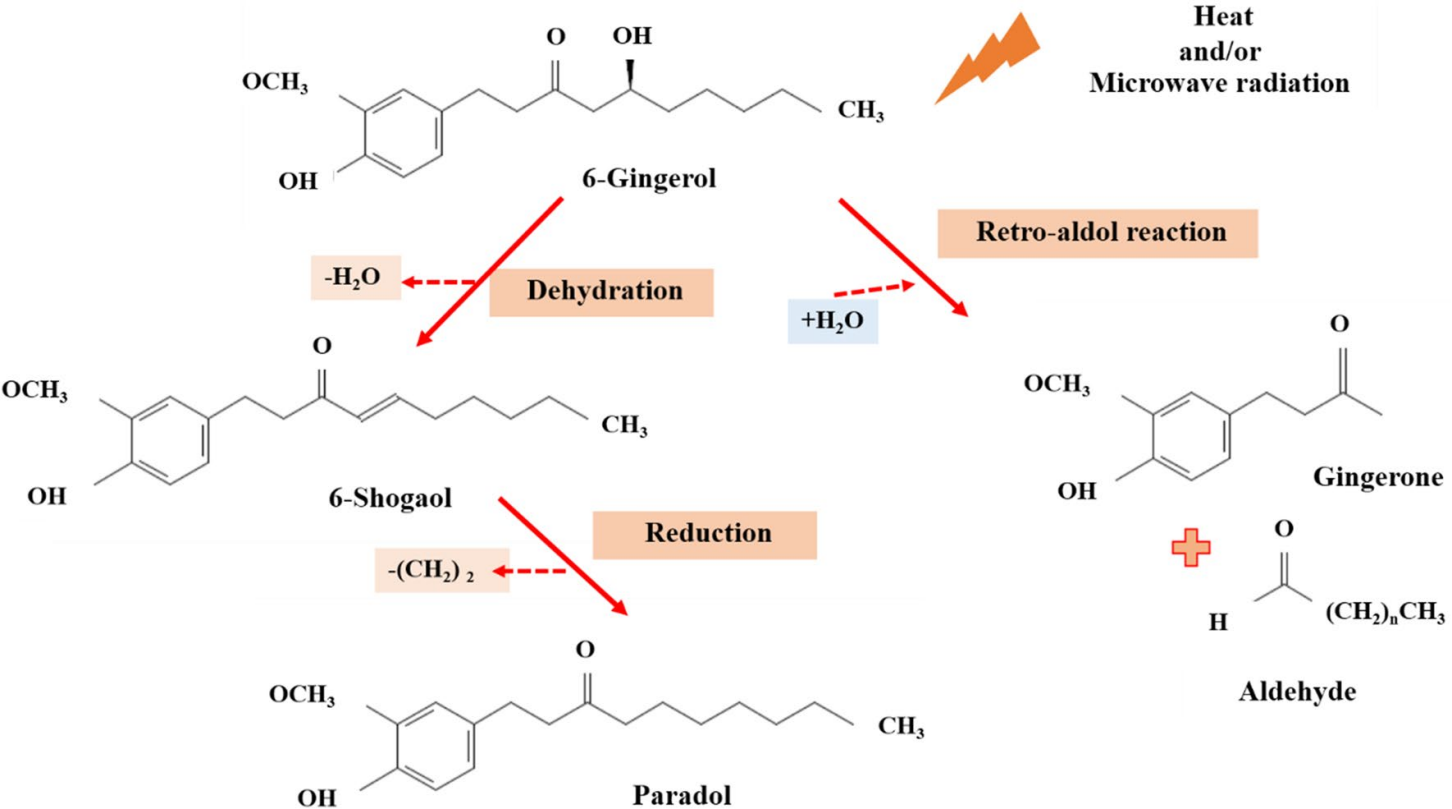
Conversion of 6-gingerol into 6-shogaol, paradol and zingerone through the heating and radiation power of the microwave process.

At high temperatures and/or high microwave power, 6-gingerol dehydrates water (H_2_O) from its structure and converts it to 6-shogaol^4^. If the reduction of (CH_2_)_2_ occurs, 6-shogaol will be transformed into paradol. In another case, microwave power promoted the retro-aldol reaction of 6-gingerol and is proposed to generate zingerone constituents with an aldehyde to deliver the products (Fig. 3). The competition of these reactions can be further demonstrated by the synthesis of 6-shogaol, paradol, and zingerone constituents. The content of 6-shogaol and zingerone was gradually increased under high microwave power (> 600 W) and a long extraction time (> 3 min). This indicates that 6-shogaol and zingerone are produced at high temperatures and high microwave power, and also by the thermal degradation of gingerol^9^. 6-Shogaol and zingerone increased on increasing the microwave power and extraction time, which resulted in increased ABTS and FRAP values. This phenomenon could be explained by 6-gingerol being dehydrated and generating H^+^ and OH^−^ radicals at high temperatures or high microwave power, resulting in the production of 6-shogaol, paradol, zingerone, and their derivatives^5,6^.

*S. mutans* is found in the oral cavity and forms a dental plaque to prevent the admission of antimicrobial agents^10^. The oral film strip is considered one of the most convenient routes for administration of these agents due to cost efficiency and ease of administration. In this study, we observed the inhibitory activity of crude ginger extract and found that the MIC and MBC of the crude ginger extract against *S. mutans* DMST 18777 were 0.5 and 31.2 mg/mL, respectively, with an inhibition zone of approximately 11.0 mm. According to the Clinical Lab Standards Institute, for an agent to be accepted as having antimicrobial capacity it must display an inhibition zone of more than 11 mm, which depends on the agent and its concentration^15^. These results agree with those reported by Mathai et al.^16^, who observed a 11.7 ± 0.6 mm inhibition zone for fresh ginger extract against *S. mutans* MTCC 497. Many studies have also shown an inhibition zone for fresh ginger extract against *S. mutans* of approximately 6–18 mm^17–19^. The incorporation of less than 16 mg/mL of crude ginger extract in the rice film was not enough to inhibit the growth of *S. mutans* DMST 18777. This might be due to the immobilization of rice molecules within the film and a high number of bacteria (2.7 × 10^6^ or 6.4 log CFU/mL) exceeding the inhibition activity^20^. The antimicrobial activity of crude ginger against *S. mutans* was 5 log CFU/mL^20^. The most efficient concentration was 3.2% (w/v) at which the inhibition zone was 12.7 ± 0.1 mm. It can be concluded that the rice-based edible film produced with 3.2% (w/v) ginger extract has the potential to be considered as an anti-caries rice film. In contrast, an edible starch film containing ginger essential oils (1–3% v/w) inhibited the growth of *Escherichia coli* with a 1–9 mm inhibition zone^21^. It seems that gram-negative bacteria (*E. coli*) are more resistant to lipophilic compounds as compared with gram-negative bacteria (*S. mutans*), which occupy a single peptidoglycan layer structure^22^.

The presence of 6-gingerol and 6-shogaol in the rice film strip could impart a pungent and aromatic taste to the film. 6-Gingerol, 6-shogaol, paradol, and zingerone can inhibit reactive oxygen species and maintain their antioxidant properties^9^. Thus, the antioxidant activity of the rice film might be related to the presence of TPC and bioactive compounds. The essential oil of crude ginger has been found to contain 44 volatile compounds^8^. In this study, the main volatile compounds in the rice film strip were α-curcumene, α-zingiberene, γ-muurolene, α-farnesene, β-bisabolene, and β-sesquiphellandrene, as identified by gas chromatography–mass spectrometry (GC–MS). These compounds are sesquiterpenes. The monoterpenes and sesquiterpenes are the main classes of volatile compounds in ginger; its antioxidant activity is attributed to α-zingiberene, camphene, α-farnesene, and β-sesquiphellandrene^23^. In this study, α-zingiberene (27.0 ± 4.3–34.6 ± 6.7%) was the main volatile compound in the rice film strip, followed by α-curcumene (13.2 ± 2.1–16.8 ± 2.9%), β-sesquiphellandrene (13.1 ± 4.5–15.3 ± 2.4%), α-farnesene (10.3 ± 1.6–12.6 ± 2.6%), β-bisabolene (6.2 ± 2.2–8.7 ± 1.0%), and γ-muurolene (6.3 ± 0.3–7.6 ± 1.2%). Wang et al.^24^ showed that α-zingiberene was the main compound of the essential oil found in ginger, its content in the range 17.4–25.4%, followed by ar-curcumene (14.1–16.4%), β-bisabolene (9.9–12.5%), and β-sesquiphellandrene (9.7–13.4%), which was consistent with this study.

## Conclusions

MAE was successfully used to extract TPC, 6-gingerol, 6-shogaol, paradol, and zingerone from dried ginger, and antioxidant efficiency was increased for a shortened extraction time by a 3^2^ full factorial design. The optimal conditions were microwave power of 400 W and an extraction time of 1 min, which showed responses close to the predicted responses. For antimicrobial activity, MIC and MBC values of 0.5 and 31.2 mg/mL, respectively, were obtained for the crude extract of dried ginger against *S. mutans* DMST 18777. Furthermore, it has been recently shown that the antimicrobial activity of rice-based edible film incorporating 3.2% (w/v) ginger extract makes the film suitable for application as an anti-caries rice film containing bioactive compounds and essential oil that can inhibit the growth of bacteria. Rice film incorporating 3.2% (w/v) ginger extract showed a significant antibacterial effect against *S. mutans* DMST 18777, proving that ginger is released from the film into the surrounding culture medium and that its antimicrobial activity has been preserved after fortification of the polymer. The presence of phenolic compounds including 6-gingerol, 6-shogaol, paradol, and zingerone, and essential oils including α-curcumene, α-zingiberene, γ-muurolene, α-farnesene, β-bisabolene, and β-sesquiphellandrene in the rice-based edible film might be helpful for several therapeutic effects. Thus, the development of a rice-based edible film incorporating dried ginger extract may constitute an alternative way of dealing with *S. mutans* resistance.

## Materials and methods

### Raw material

Fresh and 9 month-matured ginger rhizomes, grown and controlled under Good Agricultural Practice, were obtained from the Hsu Chuan Foods Co., Ltd in Chiang Rai, Thailand (www.hcgroupthailand.com). Identification was done according to a Zingiberaceae expert and the literature^25^. A voucher specimen (QBG No. 27329) was provided by the Queen Sirikit Botanic Garden (QBG), Chiang Mai, Thailand. The peeled ginger was washed in distilled water and cut into 0.2 × 4 × 0.4 cm pieces. The cut ginger was then dried using the method reported previously by Sida et al.^8^. Briefly, the ginger pieces were placed in trays and put into a hot-air dryer (Armfield, Hampshire, England) at 60 °C for 308 min. The dried ginger was ground with a hammer mill (Crompton, model 2000 Series, England) and sieved at 1.2 mm. The ginger powder was kept in an aluminum foil vacuum package and stored in a desiccator at ambient temperature for at least 24 h before further analysis.

### MAE experimental design

The MAE was carried out in a laboratory microwave (Toshiba, Model ER-300C(S) Power Max 900, frequency 2.45 × 109 Hz, Japan). A 3^2^ full factorial design was constructed to investigate the influence of two variables, microwave power (400, 600, and 800 W) and the reaction time (1, 3, and 5 min) (Table 1). The ginger powder (10 g) was added to 100 mL of 95% ethanol and extracted following the conditions represented in Table 1. The microwave-extracted samples were then filtered into a 100 mL conical flask using no. 1 Whatman filter paper. The filtrate was collected and concentrated using a rotary evaporator under vacuum at 50 ± 4 °C; finally, dry extract yield was calculated and expressed as a percentage. The dried extract samples (crude oil) were kept at 4 °C until further use.

The experimental data were analyzed using a response surface regression procedure to fit the following second-order polynomial model (Eq. 1).

1 $$ Y = \beta_{0} + \mathop \sum \limits_{i = 1}^{{}} \beta_{i} X_{i} + \mathop \sum \limits_{i = 1}^{{}} \beta_{ii} X_{i}^{2} + \mathop \sum \limits_{i = 1}^{{}} \mathop \sum \limits_{j = i + 1}^{{}} \beta_{ij} X_{i} X_{j} + e_{0} $$

where Y is the predicted response variable, $\beta_{0}$ is the constant coefficient, $\beta_{i}$ is the linear effect, $\beta_{ii}$ is the squared effect, $\beta_{ij}$ is interaction effects, and $X_{i}$ and $X_{j}$ represent the independent variables, respectively.

### Edible film preparation

The rice-based film was prepared by a casting technique according to Miksusanti et al.^21^ with some modifications. For film formation, 7 g of glutinous Thai rice powder (Newgrade, Thaiwah Co., Ltd, Thailand) was dissolved in an aqueous solution (145 mL) of water and heated at 1–2 °C/min to 80 °C on a magnetic stirrer hot plate for 1 h to promote gelatinization. At the same time, sodium carboxymethyl cellulose (0.7 g) and refined glycerine (1.75 mL) were added slowly. After entering the gelatinization stage, the ginger extract was added to the Thai rice solution to reach a final concentration of 4, 8, 16, or 32 mg/mL (0.4%, 0.8%, 1.6%, or 3.2% (w/v)), and then degassed by sonication for 30 min. The rice-based film with ginger extract was then cast in a petri dish (9 cm diameter) and dried at 50 °C for 6 h. The films were peeled off from the casing plates and conditioned for 7 days at 25 °C in a desiccator before all analysis.

### Determination of TPC

Prior to determining the TPC, antioxidant activity, and bioactive compounds, the film (10 g) was extracted by hot extraction with 10 mL of 95% ethanol at 60 °C for 24 h^26^. After that, the solution was then filtered using no. 1 Whatman paper and centrifuged at 5,500 rpm for 10 min. The TPC were examined using the method described by Singleton and Rossi^27^. The ginger solution (200 µL) and 10% Folin–Ciocalteu reagent (1 mL) were mixed and 2% (w/v) Na_2_CO_3_ was then added with a diluting solvent (water : methanol 4 : 6) to make a total volume of 10 mL. Absorbance was recorded at 740 nm after 30 min using a spectrophotometer (UV–Vis model 1601, Shimadzu, Japan).

## Antioxidant activity

### DPPH radical-scavenging activity

Extract solution (4 mL) and DPPH solution (1 mL) were mixed (0.1 mM in methanol) in a vortex mixer and then stood at room temperature in the dark for 30 min^28^. The absorbance was recorded at 517 nm. The percentage scavenging effect was calculated using Eq. 2:

2 $$ {\text{DPPH}}\;{\text{radical}}\;{\text{scavenging}}\;{\text{activity}} \left( \% \right) = \left( {\frac{{A_{0 } - A_{1} }}{{A_{0} }}} \right) \times 100 $$

where A_0_ is the absorbance of the control solution (DPPH without sample), and A_1_ is the absorbance of the ginger extract in DPPH solution.

### ABTS method

A mixture of 7 mM ABTS and 2.45 mM potassium persulphate, the ABTS solution, was stood in the dark for 14 ± 2 h before use. Afterward, the ABTS solution was diluted with ethanol to measure the absorbance of 0.7 ± 0.02 at 734 nm^29^. Ginger extract (150 µL) was allowed to react with 4,850 µL of the ABTS solution for 6 min and then read by a spectrophotometer at 734 nm.

### FRAP assay

The FRAP assay was carried out by modification of the method of Benzie and Strain^30^. The FRAP solution (3 mL) was added to 150 µL of ginger extract for 10 min at 37 °C and the absorbance was recorded at 593 nm.

## Antimicrobial activity

### Cell culture conditions

*S. mutans* DMST 18777 (ATCC 251755) was obtained from the Thailand Institute of Scientific and Technological Research. The strain was grown in Brain Heart Infusion Broth (BHI; Difco Laboratories, Sparks, MD, USA) at 37 °C for 16–24 h under anaerobic conditions.

### Evaluation of zone of inhibition

The disc diffusion method was used to determine the zone of inhibition. Paper discs impregnated with 10 µL of ginger extract and/or rice-based film (6 mm in diameter) were placed on *Mitis salivarius* agar plates which were inoculated with *S. mutans* DMST 18777 according to the standard protocol described by the National Committee of Clinical Laboratory Standards (NCCLS)^31^. The plates were incubated at 37 °C and the diameters of the inhibition zones were measured after 24 h. Filter paper discs containing DMSO without any test compounds served as a control and no inhibition was observed. Additionally, for comparative purposes, tetracycline (30 µg, 10 µL) was used as a reference standard. Each assay was performed in triplicate and repeated three times.

### Determination of MIC and MBC

The MIC and MBC of the ginger extract against *S. mutans* DMST 18777 were determined by the reference protocol of the NCCLS method^31^. The concentration of the ginger extracts used in these experiments ranged from 63 to 500 mg/mL. The density of the cell suspensions of the respective microorganisms was adjusted to 2.7 × 10^6^ CFU/mL. The suspensions were transferred onto plates and then incubated at 37 °C for 24 h. The lowest concentration which inhibited the growth of *S. mutans* DMST 18777 was taken as the MIC, while the MBC was defined as the lowest concentration that yielded no colony growth by sub-culturing on agar plates. All tests were carried out in triplicate.

### Analysis of active compounds in ginger using high-performance liquid chromatography (HPLC)

After filtering through a 0.2 µm syringe filter, the final sample (20 µL) was used for injection. Standards of 6-gingerol, 6-shogaol, paradol, and zingerone were prepared. The analysis was performed on an HPLC system (Agilent Technologies, Santa Clara, CA, USA) with a photodiode array detector. The HPLC system contained a C_18_ reverse-phase column (Water C_18_, 250 × 4.6 mm, 5 µm particle size). The gradient elution program used acetonitrile and water at a flow rate of 1.0 mL/min and detection wavelength of 282 nm. The mobile phase contained water (A) and acetonitrile (B). The gradient elution program was set as follows: from 0 to 25 min, B was isocratic at 33%; from 25 to 35 min, B was changed from 33 to 55%; from 35 to 60 min, B was changed linearly from 55 to 90%; from 60 to 65 min, B was changed linearly from 90 to 33%; and from 65 to 70 min, B was isocratic at 33%^26^.

### Headspace solid-phase microextraction (HS-SPME)

To evaluate the volatile compounds of the edible rice film incorporating crude ginger extract, the compounds were extracted using Carboxen/polydimethylsiloxane (CAR/PDMS) fiber. The sample headspace (5 g) was transferred into a 25 mL screw cap glass vial and extracted at 50 °C for 30 min. The bound volatiles were injected into GC–MS equipment^32^.

Measurement of the target analytes was performed using GC–MS (GC-17A, Shimadzu, Japan) coupled with mass spectrometry (QP 5050A, Shimadzu, Japan). A BPX-5 capillary column (30 m × 0.25 mm × 1.00 µm; SGE, Melbourne, Australia) was used for separation and run at 1.0 mL/min with helium as the carrier gas. The inlet temperature was 250 °C in split mode (1 : 50). The initial oven temperature was 80 °C for 1 min, heated to 220 °C at 5 °C/min and maintained for 10 min, and finally increased to 250 °C. The detector temperature was set at 300 °C.

### Statistical analysis

All experiments were carried out according to the relevant guidelines and regulations. Data are shown as the mean and standard deviation for triplicate analyses. Design-Expert version 6.0.10 (Stat-Ease Inc., Minneapolis, MN, USA) was applied to perform the experimental design and the data analysis. The mean comparisons of the physical and chemical properties (yield, TPC, DPPH, ABTS, FRAP, and bioactive compounds) were analyzed using ANOVA in SPSS version 17.0 (SPSS Inc., Chicago, USA). Statistical significance was analyzed at *p* ≤ 0.05 using Duncan’s multiple range tests. The graphical figures were created and modified in Microsoft PowerPoint (Microsoft Office 2013, Washington, USA).

## Data Availability

The data generated during the current study are available from the corresponding author on reasonable request.
